# Prenatal diagnosis of Aicardi syndrome based on a suggestive imaging pattern: A multicenter case‐series

**DOI:** 10.1002/pd.6085

**Published:** 2022-01-10

**Authors:** Léo Pomar, José Ochoa, Sara Cabet, Thierry A. G. M. Huisman, Dario Paladini, Philipp Klaritsch, Aurore Galmiche, Florian Prayer, Sebastián Gacio, Karina Haratz, Gustavo Malinger, Tim Van Mieghem, David Baud, Bryann Bromley, Sébastien Lebon, Estelle Dubruc, Yvan Vial, Laurent Guibaud

**Affiliations:** ^1^ Ultrasound and Fetal Medicine, Department Woman‐Mother‐Child Lausanne University Hospital and Lausanne University Lausanne Switzerland; ^2^ School of Health Sciences (HESAV) University of Applied Sciences and Arts Western Switzerland Lausanne Switzerland; ^3^ Diagnus SA Prenatal Diagnosis and Fetal Medicine Centre Córdoba Argentina; ^4^ Pediatric and Fœtal Imaging, Hôpital Femme Mère Enfant Hospices Civils de Lyon Université Claude Bernard Lyon 1 Lyon France; ^5^ Edward B. Singleton Department of Radiology Texas Children's Hospital and Baylor College of Medicine Houston Texas USA; ^6^ Fetal Medicine and Surgery Unit Gaslini Children's Hospital Genoa Italy; ^7^ Research Unit for Fetal Medicine Department of Obstetrics and Gynecology Medical University of Graz Graz Austria; ^8^ Ultrasound and Fetal Medicine Department of Obstetrics Hospital of Niort Niort France; ^9^ Department of Biomedical Imaging and Image‐Guided Therapy Medical University of Vienna Vienna Austria; ^10^ Division of Pediatric Neurology Hospital of Children Ricardo Gutiérrez Ciudad Autónoma de Buenos Aires Argentina; ^11^ Division of Ultrasound in Obstetrics and Gynecology Lis Maternity Hospital Tel Aviv Sourasky Medical Center, Sackler School of Medicine Tel Aviv University Tel Aviv‐Yafo Israel; ^12^ Fetal Medicine Unit Department of Obstetrics and Gynecology Mount Sinai Hospital and University of Toronto Toronto Ontario Canada; ^13^ Department of Obstetrics & Gynecology Massachusetts General Hospital Boston Massachusetts USA; ^14^ Pediatric Neurology Unit Department of Pediatrics Lausanne University Hospital Lausanne Switzerland; ^15^ Institute of Pathology Lausanne University Hospital Lausanne Switzerland

## Abstract

**Objectives:**

To characterize a suggestive prenatal imaging pattern of Aicardi syndrome using ultrasound and MR imaging.

**Methods:**

Based on a retrospective international series of Aicardi syndrome cases from tertiary centers encountered over a 20‐year period (2000–2020), we investigated the frequencies of the imaging features in order to characterize an imaging pattern highly suggestive of the diagnosis.

**Results:**

Among 20 cases included, arachnoid cysts associated with a distortion of the interhemispheric fissure were constantly encountered associated with complete or partial agenesis of the corpus callosum (19/20, 95%). This triad in the presence of other CNS disorganization, such as polymicrogyria (16/17, 94%), heterotopias (15/17, 88%), ventriculomegaly (14/20, 70%), cerebral asymmetry [14/20, 70%]) and less frequently extra‐CNS anomaly (ocular anomalies [7/11, 64%], costal/vertebral segmentation defect [4/20, 20%]) represent a highly suggestive pattern of Aicardi syndrome in a female patient.

**Conclusion:**

Despite absence of genetic test to confirm prenatal diagnosis of AS, this combination of CNS and extra‐CNS fetal findings allows delineation of a characteristic imaging pattern of AS, especially when facing dysgenesis of the corpus callosum.

## Key points

What is already known about this topic?

- Aicardi syndrome (AS) is a rare developmental encephalopathy, characterized by the classic triad of infantile spasms, agenesis of the corpus callosum, and chorioretinal lacunae
- As the genetic etiology of AS is unknown and its classic triad cannot be extrapolated to prenatal diagnosis, to establish a prenatal imaging pattern is of major interest

What does this study add?

- We confirmed and completed a prenatal imaging pattern highly suggestive of AS, based on the most frequent features encountered in a large series: arachnoid cysts associated with a distortion of the interhemispheric fissure, agenesis of the corpus callosum, cortical malformations and heterotopias, and less frequently extra‐CNS anomaly (ocular anomalies, costal/vertebral segmentation defect)

## 1 INTRODUCTION

Aicardi syndrome (AS) is a rare developmental encephalopathy, characterized by the classic triad of infantile spasms, agenesis of the corpus callosum (ACC), and chorioretinal lacunae, as initially described by Jean Aicardi in 1965.^1^ Its prevalence is estimated between 1/93,000 and 1/167,000 living births,^2^ mainly affecting females, with few cases described in Klinefelter patients (47,XXY).^3^, ^4^ An X‐linked dominant transmission has been postulated, but the exact genetic etiology remains unknown.^5^, ^6^

Currently, diagnosis of AS is based on a combination of clinical and imaging features. In addition to the classic triad, major features have been described, mainly using cerebral MR imaging (MRI): cortical malformations (polymicrogyria), periventricular and subcortical heterotopias, arachnoid cysts around the third ventricle, choroid plexus cysts, and optic nerve/disc coloboma or hypoplasia. Additional features can also be suggestive of the diagnosis especially when the classic triad is incomplete: cerebral asymmetry, vertebral and rib malformations (hemivertebrae, bifid ribs), microphthalmia, vascular malformations, split‐brain EEG (asynchronous multifocal epileptiform discharges with interhemispheric dissociation), and facial dysmorphism including prominent premaxilla with upturned nasal tip and sparse lateral eyebrows.^6^

Diagnosis of AS based on the classic triad cannot be extrapolated to prenatal diagnosis as seizures and chorioretinal lacunae cannot reliably be diagnosed before birth. Indeed, AS can only be suspected if major or/and additional features are seen in a female fetus who has normal genetic testing results. Therefore, AS is rarely diagnosed prenatally and only a small number of cases have been reported.^7^ Our goal was to confirm and complete a prenatal imaging pattern suggestive of the diagnosis, based on the most frequent prenatal imaging features encountered in a large series of AS cases suspected prenatally with postnatal confirmation.

## 2 METHODS

### 2.1 Study settings and participants

This study is a retrospective analysis of cases of AS prenatally suspected after a dedicated prenatal imaging workup, including sonographic and MRI exams, performed in tertiary centers, over a 20‐year‐period (2000–2020) and pathologically/postnatally confirmed. Cases were collected from the following centers: Lausanne University Hospital (Lausanne, Switzerland), HFME (Lyon, France), Lis Maternity Hospital (Tel Aviv, Israel), Diagnus (Córdoba, Argentina), Hospital of Children Ricardo Gutiérrez (Buenos Aires, Argentina), Medical University of Vienna and Medical University of Graz (Austria), Mount Sinai Hospital (Toronto, Canada), Gaslini Children's Hospital (Genoa, Italy), Texas Children's Hospital (Houston, USA), Massachusetts General Hospital (Boston, USA). The study was approved by the local Institutional Review Boards according to each country's requirements.

### 2.2 Inclusion and exclusion criteria

Criteria for inclusion in our study were the following: (1) Suspicion of AS after a prenatal evaluation in a tertiary center; (2) Prenatal and pathological/postnatal data and imaging available for review; (3) Pathological/post‐natal confirmation of AS based either on presence of the classic triad (corpus callosum agenesis, infantile spasms, distinctive chorioretinal lacunae) or presence of distinctive chorioretinal lacunae associated with another anomaly of the classic triad plus at least two other major or additional features either at autopsy or during clinical work‐up^8^, ^9^; (4) Negative results following infectious and genetic etiological work‐up.

Cases were excluded from the analysis if (1) Etiological work‐up found another diagnosis; (2) Pathological or postnatal confirmation of AS diagnosis was not available; (3) Assessment or imaging quality do not correspond to guidelines for dedicated neurosonography and fetal MRI at the time of the examination (ISUOG guidelines^10^, ^11^ for cases included after 2007 and 2017, respectively); (4) Prenatal imaging not performed in a referral center; (5) Documented refusal to use clinical and imaging data for research.

### 2.3 Data collection

Anonymous clinical data and prenatal/postnatal imaging were collected by the principal investigators (Léo Pomar and Yvan Vial), through a datasheet via the secure messaging system of Lausanne University Hospital. Each referent kept the source file allowing to link the identity of the patients of their center with the anonymized number used for the study.

### 2.4 Analysis of imaging data

Prenatal sonographic and MR iconography was reviewed (Léo Pomar, Yvan Vial, and Laurent Guibaud) to identify features associated with AS. The quality of the examinations was evaluated based on ISUOG guidelines.^10^, ^11^, ^12^ Ultrasound examinations were considered to be of “good” quality when the trans‐vaginal or trans‐abdominal high‐frequency approach was performed using axial, sagittal and coronal sections of the fetal brain, sections of the eyeballs, optic nerve and retina, with good illustration of the anomalies. Quality was considered “sufficient” if the iconography included an examination of the brain in three orthogonal planes, as well as an examination of the eyeballs with good illustration of the anomalies found, but without detailed description of the eyes (optic disc, retina, optic nerve). The quality was considered “insufficient” when appropriate examination of the brain or of the eyes was not available.

MRIs were considered of “good” quality if a three‐plane examination of the fetal brain, eyeballs, optic nerves and retina was available in at least one T2 weighted‐contrast sequence (other sequences examined: T1 weighted‐contrast, single‐shot high resolution, diffusion weighted or tensor imaging). MRIs were considered of “sufficient” quality if a three‐plane examination of the fetal brain and eyeballs was available. MRI scans were considered to be of “insufficient” quality if three‐plane fetal brain and/or eyeball scans were not available.

## 3 RESULTS

### 3.1 Study participants

Between 2000 and 2020, 28 cases of AS were suspected based on prenatal imaging from the 12 international centers involved in our study. Pathological/post‐natal confirmation of the diagnosis was available for 20 cases (71.4%). The eight unconfirmed cases included seven patients lost to follow‐up after prenatal diagnosis, and one case in which a final diagnosis of oculo‐cerebro‐cutaneous syndrome was established after birth.

The 20 cases included had pathological/postnatal confirmation of the diagnosis based on the presence of the classic triad (ACC, distinctive chorioretinal lacunae, infantile spasms) or at least presence of chorioretinal lacunae associated with another anomaly of the classic triad and at least two other major or accessory features.

Fourteen patients (70%) were primigravida whereas six were multigravida, of whom three (15%) had one or more miscarriages prior to this pregnancy, without any personal or familial history of congenital anomalies. One patient (Case 6) had a periconceptional cytomegalovirus seroconversion for which congenital infection was ruled out by negative PCR in amniotic fluid and neonatal urine. Apart from one case with nuchal translucency >95th percentile at 12 weeks of gestation (w), no abnormality was detected by first trimester ultrasound or serum markers (PAPP‐A and bHCG after 2009, AFP oestriol and HCG in the second trimester before 2009).

### 3.2 Prenatal imaging

All included patients were referred for suspected corpus callosum anomalies and/or cerebral cysts and/or fetal ventriculomegaly in the second or third trimester. The median gestational age at the time of the referral ultrasound was 23w (18 to 37w). Fifteen patients underwent fetal brain MRI, performed at a median gestational age of 31w (22 to 36w). Description of MR protocols, as well as data on prenatal sonographic and MR examination quality, are presented in Table S2.

### 3.3 Prenatal imaging features

#### 3.3.1 Cerebral cysts

**FIGURE 1:**
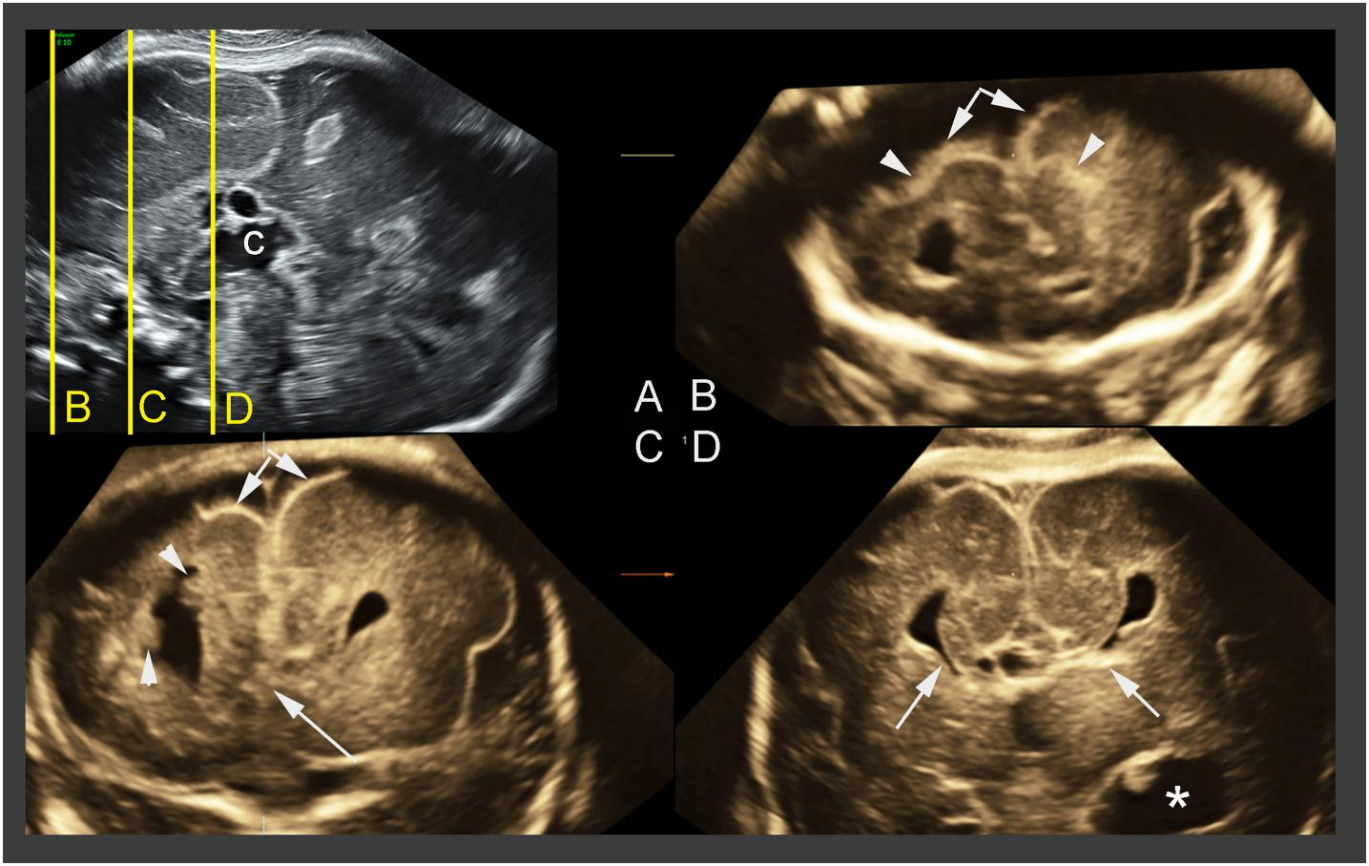
Aicardi syndrome proposed diagnostic triad: corpus callosum agenesis, inter‐hemispheric cyst and distortion of the inter‐hemispheric fissure. Case 8: Transvaginal multiplanar three‐dimensional imaging in a 28 weeks old female fetus. (A) Reference midsagittal view. On this, complete agenesis of the corpus callosum and one of the interhemispheric cysts are visible. The yellow lines correspond to the coronal planes depicted in B–D; (B) trans‐frontal plane showing the asymmetry of the two hemispheres (arrows) and abnormal sulcation (arrowheads); (C) on another trans‐frontal plane just posterior to the previous one, it is possible to confirm the hemisphere asymmetry (double arrows). In addition, moderate distortion of the interhemispheric fissure (single arrow) and two hyperechoic nodules of subependimal heterotopia (arrowheads) are visible; (D) transcaudate plane demonstrating the typical bullhorns splaying of the frontal horns, typical of complete agenesis of the corpus callosum (arrows) and dilatation of a temporal horn of the ventricle (asterisk), consistent with the severe ventriculomegaly (20 mm) evident on axial and transcerebellar coronal view (not shown) (courtesy of Dario Paladini)

**FIGURE 2:**
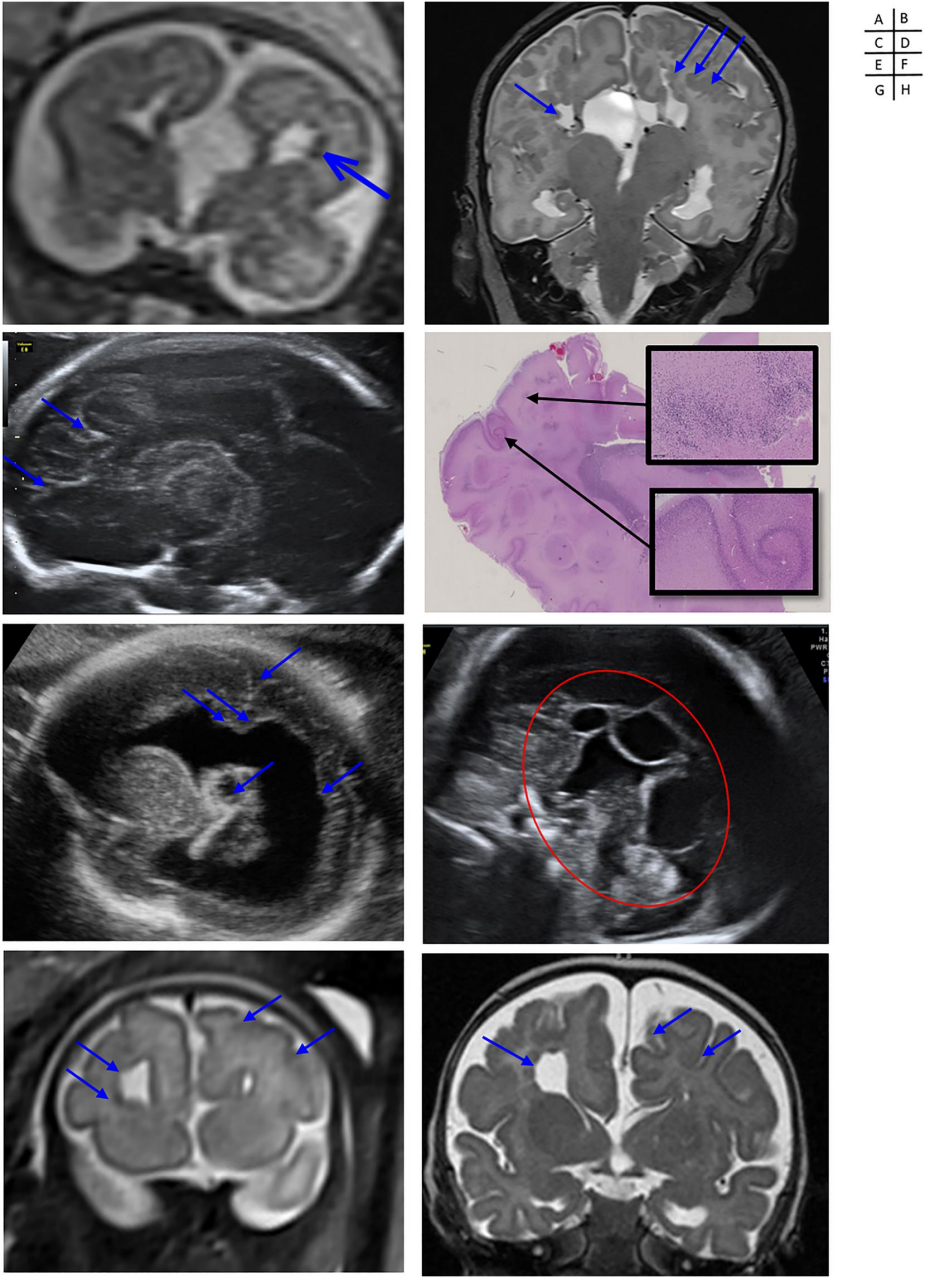
Prenatal/postnatal imaging and pathological aspect of cortical abnormalities associated with Aicardi Syndrome. Case 2: (A) Prenatal (T2w, coronal, 25w) and (B) postnatal MRI (T2w, coronal) showing complete agenesis of the corpus callosum, with interhemispheric cysts, distorsion of the interhemispheric fissure, and subependymal, periventricular and cortico‐subcortical heterotopias (courtesy of Yvan Vial and Sébastien Lebon). Case 3: (C) US (parasagittal section at 22w) and (D) giant and X10 histo‐pathological sections (hematoxylin and eosin stain) of the left parietal lobe showing an association of polymicrogyria and heterotopias (courtesy of Léo Pomar and Estelle Dubruc). Case 12: (E) US (parasagittal, 24w) showing severe unilateral ventriculomegaly with periventricular heterotopias, choroid plexus cyst and polymicrogyria(courtesy of José Ochoa). Case 13: (F) US (medio‐sagittal section at 31w) showing a complete agenesis of the corpus callosum associated with juxtaposed interhemispheric cysts (courtesy of José Ochoa). Case 16: (G) Prenatal (T2w, coronal, 33w) and (H) postnatal MRI (T2w, coronal) showing complete agenesis of the corpus callosum associated with a distorsion of the interhemispheric fissure, polymicrogyria (on the side of the enlarged frontal horn) and periventricular heterotopias (arrows) (courtesy of Thierry A. G. M. Huisman)

Extra‐axial cysts were present in all cases (Table S1, Figures 1 and 2) located within the interhemispheric fissure in parieto‐frontal or parieto‐occipital location (including cysts of the quadrigeminal cistern) in 12 (80%; Cases 1, 2, 3, 4, 5, 8, 9, 11, 12, 13, 14, 15, 16, 17, 18, and 20) and three (15%, Cases 6, 10, and 19) cases respectively, and within the posterior fossa in three cases (15%, Cases 1, 5, and 14). Cysts were either single (12/20, 60%) or multiple either at the same or at different locations in five (25%; Cases 1, 4, 12, 13, and 18) and four (15%; Cases 1, 5, 14, and 18), respectively. In all cases, cysts were associated with mass effect on the adjacent parenchyma.

Choroid plexus cysts were described in three cases (15%; Cases 9, 12, and 18). In one case, the choroidal plexus cyst was unusually large and associated with a solid component demonstrating high Doppler signal, suggestive of choroid plexus papilloma.

#### 3.3.2 Distortion of the anterior part of the interhemispheric fissure

Distortion of the interhemispheric fissure was demonstrated in all cases (20/20), giving an asymmetrical aspect of the frontal lobes in 14 cases (70%).

#### 3.3.3 Agenesis of the corpus callosum

ACC was diagnosed in all cases, except one (19/20, 95%, Figures 1 and 2), and was demonstrated as complete or partial in 17 and 2 cases, respectively, involving the splenium in Case 6 and genu and rostrum in Case 3. Discordance between ultrasound and MRI was observed in three cases (Case 3: partial agenesis vs. complete agenesis; Case 6: partial agenesis vs. complete corpus callosum; Case 16: complete agenesis vs. complete corpus callosum), mainly related to difficulties in midline analysis, due to interhemispheric cyst mass effect. Identification of Probst bundles associated with ACC was demonstrated when DTI sequences were used (Cases 2, 11, and 18).

#### 3.3.4 Cortical and migration abnormalities (Figures 1 and 2)

Cortical anomalies were demonstrated, mainly in cases investigated after 2007 (16/17, 94%), since in the three older cases the imaging quality precluded detection of such anomalies. Diffuse versus focal polymicrogyria was shown in three (2, 14, 15) and 13 cases respectively, predominantly in frontal location, with unilateral involvement in six cases.

Neuronal migration abnormalities were observed in 15 cases (15/17, 88%; Cases 1, 2, 3, 4, 5, 6, 7, 8, 9, 15, 16, 17, 18, 19, and 20). Heterotopia and nodules were located in the periventricular (10/13, 77%), subependymal (6/13, 46%), and subcortical areas (4/13, 31%). Heterogeneous aspect of the basal ganglia was noted in three cases (Cases 2, 3, and 8), consistent with heterotopias. Schizencephaly was suspected in four cases (20%). In Cases 2 and 3, closed‐lip communication between pericerebral space and ventricles, bordered by polymicrogyria, was suggestive of a true schizencephaly reflecting an early ischemic lesion, whereas in Cases 14 and 17, the aspect was related to extreme thinning of the parenchymal mantle facing unilateral severe ventriculomegaly leading to near‐communication between the ventricular lumen and the subarachnoid space.

In four cases (Cases 11, 15, 16, and 17), MRI diagnosed cortical abnormalities which were not described on ultrasound, and in three other cases (Cases 2, 3, and 14) MRI confirmed suspected heterotopias sonographically described as “hyperechoic foci.”

#### 3.3.5 Ventriculomegaly

Unilateral versus bilateral ventriculomegaly were observed in nine (Cases 1, 2, 3, 6, 12, 13, 14, 16, and 17) and five (Cases 5, 7, 8, 9, and 11) cases, respectively (total 14/20, 70%). These ventriculomegalies were severe in eight cases (>15 mm, 57%). On ultrasound, a thickened, hyperechoic and heterogeneous ventricular wall was described in several cases (Cases 2, 3, and 8), suggesting periventricular migration disorders. On MRI, occipital and frontal periventricular irregular hyperintensities and nodules were described in these cases, also suggesting heterotopias.

#### 3.3.6 Posterior fossa anomalies

Posterior fossa anomalies were found in seven cases (35%). Cases 1, 5, 14, and 18 presented a cystic malformation of the posterior fossa: arachnoid cyst in three cases and a Blake's pouch cyst in the last one. An enlarged cisterna magna was found in case 16. Cases 5, 11, 14, and 15 showed asymmetry of the cerebellar hemispheres, with abnormal foliation of the inferior border of the left cerebellar hemisphere in Case 5, and unilateral hypoplasia of one hemisphere in Cases 14 and 15.

#### 3.3.7 Ocular anomalies

Ocular anomalies were identified mainly in the most recent cases displaying high prenatal imaging quality and providing detailed analysis of the globes, optic nerves and retinas (11 cases).

**FIGURE 3:**
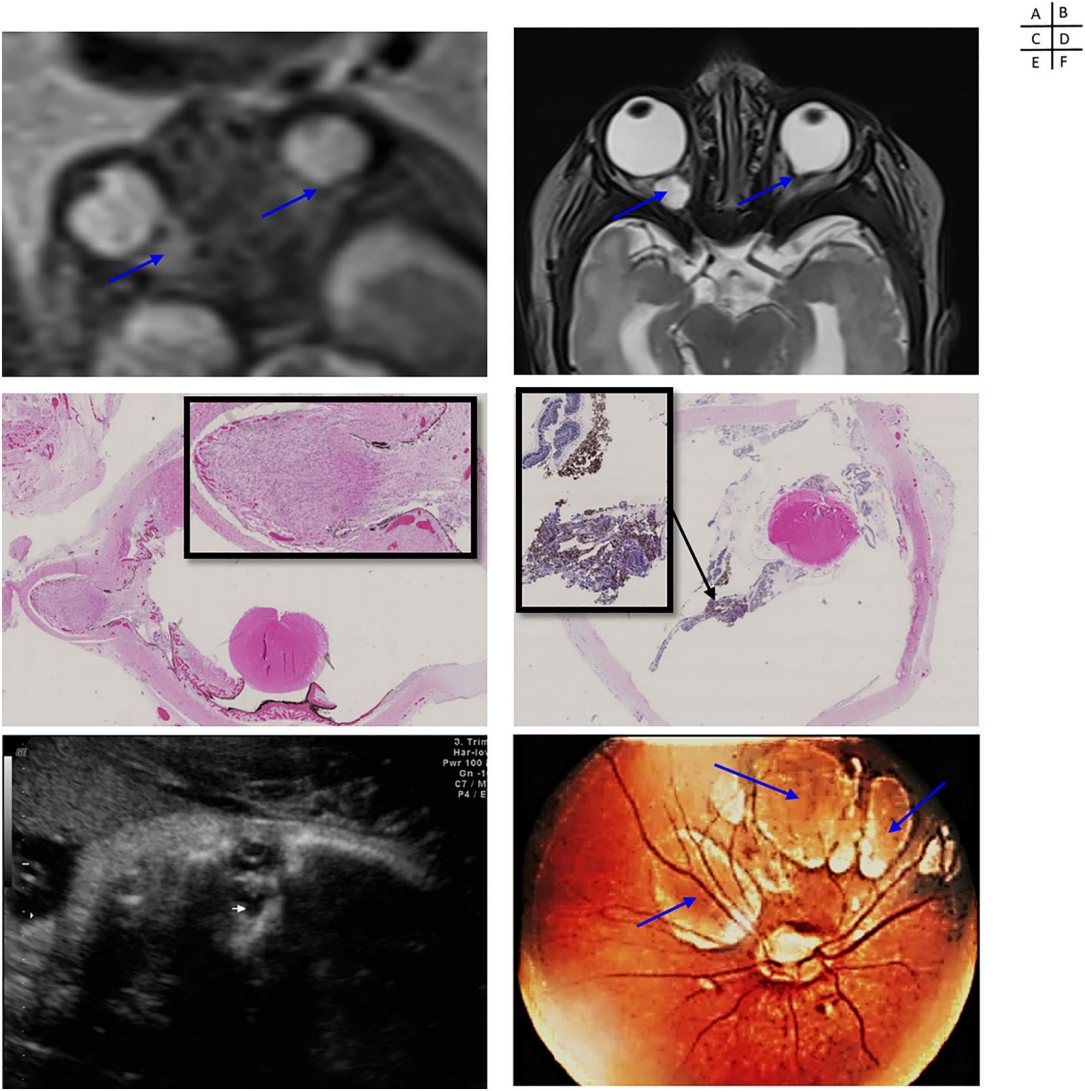
Prenatal imaging, pathology and funduscopy of ocular anomalies associated with Aicardi Syndrome. Case 2: (A) Prenatal (T2w, axial, 25w) and (B) postnatal MRI (T2w, axial) showing unilateral microphthalmia and bilateral colobomas on retina and optic nerve (courtesy of Yvan Vial and Sébastien Lebon). Case 3: X10 and X20 histo‐pathological sections (hematoxylin and eosin stain) of the eyeball showing a colobomatous lesion of the retina (C) and a chorioretinal lacunae with hyperpigmented foci (D) (courtesy of Estelle Dubruc). Case 12: (E) US (para‐sagittal, 24w) aspect of a microphthalmia with retinal coloboma (courtesy of José Ochoa). Case 19: (F) Funduscopy with microphthalmia and chorioretinal lacunae in the macula (courtesy of Léo Pomar)

Of these cases, nine had abnormalities (9/11, 82%; Cases 1, 2, 3, 4, 6, 12, 15, 18, and 20). Microphthalmia was found in seven cases (Cases 1, 2, 4, 12, 18, 19, and 20), unilateral in six and bilateral in one. Retinal or optic nerve colobomas were found in seven cases (Cases 1, 2, 3, 6, 12, 15, and 18), described as bilateral/unilateral in one and six cases, respectively (Figure 3). Microphthalmia was detected on both ultrasound and MRI, however MRI was able to identify optic nerve coloboma, described as a “bulge” proximal to the retina. The retinal colobomas appeared as “cracks” on sagittal and axial sections of the eyeball. On ultrasound, the appearance of such coloboma was similar to the one demonstrated by MR.

#### 3.3.8 Other fetal anomalies

**FIGURE 4:**
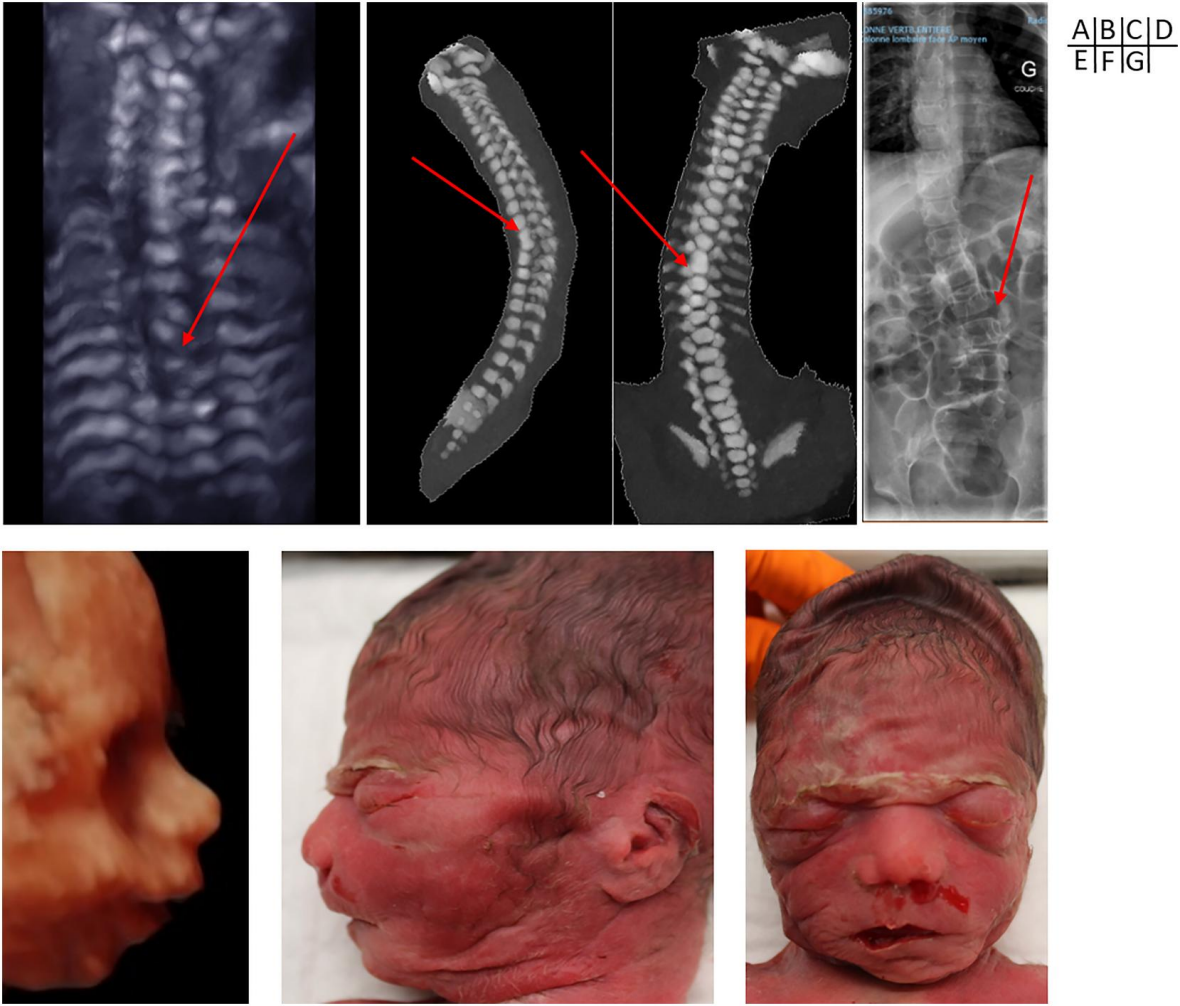
Prenatal imaging and postnatal appearance of other anomalies associated with Aicardi syndrome (A–D) Prenatal ultrasound (3D bone rendering), prenatal computed tomography, and postnatal X‐rays showing thoracic scoliosis with hemi‐vertebrae (A and D) or vertebral bloc (B and C) in Cases 2, 5, and 19 (courtesy of Léo Pomar and Laurent Guibaud) (E–G) Prenatal ultrasound (3D surface) and post‐mortem aspect showing facial dysmorphism (bulging premaxilla, upturned nostrils, retrognathia) in Case 3 (courtesy of Léo Pomar and Estelle Dubruc)

Two fetuses (Cases 2 and 6) had in utero repeated spastic movements, suggestive of seizures. Vertebral anomalies were identified in four cases (20%) (Cases 3, 5, 9, and 20). These anomalies were scoliosis with thoracic (Cases 3, 5, and 20) and lumbar (Case 9) hemi‐vertebrae (Figure 4).

Of the examinations providing 3D imaging of the fetal face, dysmorphism consistent with the one described in ASs was identified in four cases (4/10, 40%) (Cases 1, 3, 4, and 6) (Figure 4).

The choroid plexus cyst with extensive vascularization (Case 18) strongly suggested a choroid plexus papilloma, a tumor described in AS.

### 3.4 Confirmation of AS diagnosis on pathological/post‐natal data

Pathological and postnatal features are summarized in Table 1. Pathological examination or postnatal imaging provided additional information in 40% (8/20) of cases, mainly on the description of cortical and spinal anomalies.

**TABLE 1:** Synthesis of prenatal and postnatal classic triad, major and supporting features associated with Aicardi syndrome

Aicardi syndrome features		Prenatal, *n*		Postnatal, *n*	
Classic triad	Dys/a‐genesis of corpus callosum	19/20	(95%)	19/20	(95%)
	Distinctive chorioretinal lacunae	NA		20/20	(100%)
	Spasms	02/20	(10%)	14/15	(93%)
Major features	Cortical malformations (polymycrogyria, pachgyria, agyria)	16/17	(94%)	18/20	(90%)
	Periventricular and subcortical heterotopia	15/17	(88%)	18/20	(90%)
	Inter‐hemispheric cysts	20/20	(100%)	20/20	(100%)
	Optic disc/nerve coloboma or hypoplasia	7/11	(64%)	11/20	(55%)
	Distorsion of the inter‐hemispheric fissure	20/20	(100%)	20/20	(100%)
Supporting features	Ventriculomegaly (>10 mm)	14/20	(70%)	14/20	(70%)
	Cerebral hemispheric asymmetry	14/20	(70%)	16/20	(80%)
	Microphtalmia	7/11	(64%)	9/20	(45%)
	Posterior fossa abnormalities	7/20	(35%)	7/20	(35%)
	Vertebral and costal abnormalities	4/20	(20%)	7/20	(35%)
	Vascular malformations or tumor	1/20	(5%)	1/20	(5%)
	Facial dysmorphic features	4/10	(40%)	20/20	(100%)

Six pregnancies (30%) were terminated on parental request because of poor expected post‐natal neurodevelopmental outcome. Both brain parenchyma and retinal fetal examination confirmed the diagnosis in all cases. Indeed, pathological examination confirmed chorioretinal lacunae in all cases (Figure 3). Macroscopic examinations confirmed microphthalmia in four cases, described as unilateral in three and bilateral in one case, respectively. Microscopic examination of the eyes confirmed the presence of colobomas of the retina in four cases, described as unilateral in three and bilateral in one case, respectively, including two cases with colobomas at the insertion of the optic nerve. Pathological examination of Case 9 found bilateral optic nerve colobomas, not suspected on prenatal imaging. Pathological examination of Case 3 revealed ectopic pigmented retinal epithelial cells and foci of retinal hyperpigmentation in addition to colobomatous defects. Pathological examination of this latter case showed partial ACC (discordant with prenatal imaging data). Pathological examination of Case 18 identified unilateral agenesis of the 12th rib, which was not prenatally diagnosed. In the remaining cases, pathological examination confirmed the prenatal imaging data.

Fourteen fetuses were born at term. All had chorioretinal lacunae on fundoscopy (Figure 3), which confirmed the prenatal diagnosis of AS.

#### 3.4.1 Fundoscopy

Chorioretinal lacunae in the papilla or macula were found in all cases, described as bilateral/unilateral in 10 (Cases 2, 5, 7, 10, 13, 14, 15, 16, 17, and 20) and four (Cases 6, 8, 12, and 19) cases respectively. Microphthalmia was found in five cases (35%, cases) (Cases 2, 12, 17, 19, and 20). Retinal or optic nerve colobomas were found in seven cases (50%) (Cases 2, 6, 8, 12, 14, 15, and 16), described as bilateral and unilateral in four (Cases 2, 14, 15, and 16) and three (Cases 6, 8, and 12) cases respectively. Discrepancy with prenatal examinations suggestive of none anomalies or unilateral ocular anomaly that turned out to be present or bilateral postnatally, was found in three cases.

#### 3.4.2 Electroencephalograms (EEGs)

In the seven cases in whom data were available, EEG was abnormal. Cases 2, 14, and 16 showed abundant multifocal epileptiform discharges with asymmetric background activity and epileptic spasms on neonatal examination. Case 2 had focal epilepsy with impaired awareness and unstructured background activity. For the three remaining cases, no precise data regarding EEGs exam could be collected.

#### 3.4.3 Imaging

Postnatal MRI was performed for all newborns and confirmed prenatal imaging in all cases, providing additional information in six cases (including hemispheric asymmetry, polymicrogyria, subependymal heterotopias, mega cisterna magna, optic nerve hypoplasia, hippocampal malrotation).

#### 3.4.4 Neurologic symptoms and development

For all live births, clinical data could be collected at different stages of life (from one month to 12 years). Epilepsy or spasms were present in 13 of them (93%), described as drug‐resistant in four cases. In addition, 10 children (66%) who were examined during childhood, had severe neurodevelopmental delay (examination from 3 months to 12 years of age [Cases 2, 5, 7, 8, 12, 13, 14, 16, 17, and 20]). After the first year of life, the majority of children had multiple disabilities (spastic, tetra‐paresis, severe paralysis, and hypertonia). Two presented with digestive complications (14%).

#### 3.4.5 Vertebral anomalies

Vertebral anomalies were identified in six cases (43%) (Cases 2, 5, 13, 14, 17, and 20) including scoliosis with thoracic and lumbar hemivertebrae.

#### 3.4.6 Genetics data

Karyotyping and CGH array, performed in 19 and five cases respectively, identified no chromosome number abnormalities nor abnormal copy number variations. Exome sequencing, performed in the five most recent cases, found no known pathogenic variants.

## 4 DISCUSSION

The imaging findings reported in our series are in concordance with those previously described in prenatal reports and post‐natal series of AS. Hopkins et al. reported cerebral MRI findings of 23 patients with AS. All had corpus callosum agenesis (partial or complete), polymicrogyria (predominantly in the frontal and perisylvian areas) and heterotopias (periventricular, in the corona radiate and cerebellar). The majority of patients (95%) had also intra‐cranial cysts, mainly interhemispheric, and fossa posterior anomalies.^13^ The frequency of cerebellar anomalies was higher in their series, two third and half of cases presenting dysplastic/hypoplastic cerebellar hemispheres and enlarged cisterna magna, respectively. In the other series, the frequency of posterior fossa anomalies was close to our personal data (6%–63%).^14^, ^15^, ^16^, ^17^, ^18^ The localization and description of intra‐cranial cysts in our series was consistent with previous literature data, with cysts located predominantly in the interhemispheric fissure in the frontal area, or between occipital lobes or, to a lesser extent, in the posterior fossa.^13^, ^18^, ^19^ These cysts correspond to type 2b (glioependymal, associated with heterotopia, and polymicrogyria) and 2d (arachnoid cysts with CSF‐like signal), according to the classification of Barkovich et al.^20^ Distortion of the interhemispheric fissure was present in most of the previously published cases, but this feature was rarely emphasized by the authors, preferring to highlight an asymmetry of the cerebral hesmispheres. Although this feature is not specific to AS,^21^ this distortion is important to underline, as an easily accessible feature since interhemispheric fissure is included in the analysis of the anterior complex when performing routine sonographic exam.^22^ Frequency of cortical/migration anomalies is close to 100% in all series, including mainly nodular and diffuse heterotopia and polymicrogyria.^13^, ^18^ In cases 3, 11, and 20, prenatal imaging identified dysgyria or delayed sulcation, which was related to polymicrogyria on postnatal imaging or autopsy. Since postnatal imaging offers more details on cortical anomalies than prenatal imaging, one should note that polymicrogyria may vary from focal to diffuse forms but was more frequently focal in our study. Therefore, when AS is prenatally suspected, one should examine closely to the cortical ribbon to look for any focal cortical dysplasia, which can be easily overlooked. Use of DTI in Case 8 revealed disruption of cortical white matter organization close to polymicrogyria, which has been reported as a specific feature of AS by Wahl et al. in two patients.^23^ In some patients, we also reported nodular heterotopia of the basal ganglia area, found in up to 76% of cases in postnatal series.^24^

In our series, distinctive chorioretinal lacunae were confirmed in all cases but we may have overestimated the frequency of this anomaly, as it was a nonexclusion criterion. These lacunae were present in 88%–100% of cases in other series.^6^, ^25^ Chorioretinal lacunae associated with hyperplasia of pigment epithelium, found in post‐mortem examination of our terminated fetuses, were very close to those described by Del Pero et al.^26^ Since it is not possible to diagnose chorioretinal lacunae, prenatal ultrasound and MRI should focus on other ocular features, including microphthalmia and optic nerve or disc colobomas, which were both reported in 17%–45% and in 55%–92% of cases, respectively in the literature.^6^, ^18^, ^25^ Although microphthalmia is commonly detected by prenatal imaging, coloboma is more difficult to diagnose using prenatal MRI, as illustrated by the low rate of detection in our series (64%), and also as reported in the literature.^18^ Finally, optic nerve hypoplasia, described on postnatal MRI in high proportion (78%) by Masnada et al. was found only in seven of our 20 prenatal cases (35%).

Dysmorphic features may be difficult to identify on prenatal imaging. We identified prenatal dysmorphic features in 40% of cases although a distinctive dysmorphism after birth was present in all cases. Indeed, the sagittal view of the profile can only identify the prominent maxilla described in AS. The use of 3D imaging may lead to a better identification of the other dysmorphic features: upturned nasal tip and upper lip, micrognathia and sparse lateral eyebrows (Figure 4).^8^, ^27^

In keeping with our main objective, based on the most frequent prenatal imaging findings encountered in our series, the most suggestive prenatal imaging pattern is represented by a triad, which includes one or more extra‐axial cysts associated with distortion of the interhemispheric fissure and partial or complete ACC in a female fetus. This diagnostic triad can be reinforced by major CNS organization anomalies, such as cortical/migration anomalies, or by ocular anomalies and less frequently vertebral anomalies. Regarding the ophthalmological anomalies, one should note that the pathognomonic chorioretinal lacunae, found in all cases of our series on post‐natal fundoscopy, could not be diagnosed on prenatal imaging. Therefore, in the absence of both genetic testing and prenatal access to chorioretinal lacunae, our prenatal imaging pattern based on the previously described features, is of major interest to highly suggest the diagnosis of AS in the female fetus, especially when dealing with prenatal counselling of ACC due to the poor cognitive and ophthalmological prognosis of this condition.

Finally, we should underline the strength and the main limitations of our study. Indeed, this series represent the largest one of prenatally suspected cases of AS, based on the recruitment of international referral centers, which were expected to provide high quality examinations. The main limitation is the study period of 20 years, which was required to collect a large number of cases due to the rarity of this condition, and which obviously introduced some bias especially related to the evolution of imaging and genetic testing over time. Indeed, cases prior to 2010 were mainly studied by karyotype and, those between 2010 and 2017, by array‐CGH, due to difficult access to WES. Array‐CGH can detect deletions and duplications associated with other syndromes that have some features which mimic those of AS,^14^, ^28^ and WES can detect gene variants associated with conditions that have similar corpus callosum and cortical anomalies such as tubulinopathies, which may represent another differential diagnosis,^29^, ^30^ even if cyst are not present in this condition. Thus, we cannot exclude that some of the older cases included could result from another genetic cause not detected by karyotype. However, all included cases had distinctive chorioretinal lacunae, described as a pathognomonic sign of AS.^31^ However, chorioretinal lacunae and colobomas are also described in oro‐facio‐digital 9 syndrome, but interhemispheric cysts are not described in this syndrome and we did not observe digital anomalies in our cases.^32^ We should also underline that the small proportion of patients who underwent WES in our study did not provide any new highlights on the genetics of AS.^5^, ^33^, ^34^, ^35^ The other bias related to the long study period is the heterogeneous quality of prenatal imaging, overlooking subtle prenatal features in older cases, especially regarding cortical and ocular anomalies.

## 5 CONCLUSION

Finally, despite the lack of a genetic test to enable a prenatal diagnosis of AS, prenatal imaging can identify a combination of CNS and extra‐CNS features which form a characteristic imaging pattern of this syndrome, especially when facing intra‐cranial cyst associated with distortion of the inter‐hemispheric fissure and callosal dysgenesis.

## CONFLICT OF INTERESTS

All authors have completed the ICMJE disclosure form at www.icmje.org/coi_disclosure.pdf and have no conflicts of interest to declare.

## Supporting information

Supporting Information S1

Supporting Information S2

## Data Availability

All data used are presented in the supplementary tables.
